# A Proof-of-Concept Study on the Therapeutic Potential of Au Nanoparticles Radiolabeled with the Alpha-Emitter Actinium-225

**DOI:** 10.3390/pharmaceutics12020188

**Published:** 2020-02-21

**Authors:** Evangelia-Alexandra Salvanou, Dimitris Stellas, Charalampos Tsoukalas, Barbara Mavroidi, Maria Paravatou-Petsotas, Nikolaos Kalogeropoulos, Stavros Xanthopoulos, Franck Denat, Gautier Laurent, Rana Bazzi, Stephane Roux, Penelope Bouziotis

**Affiliations:** 1 Institute of Nuclear & Radiological Sciences & Technology, Energy & Safety, National Center for Scientific Research “Demokritos”, 15341 Athens, Greece; salvanou@rrp.demokritos.gr (E.-A.S.); ctsoukal@rrp.demokritos.gr (C.T.); mparavatou@rrp.demokritos.gr (M.P.-P.); staxan@rrp.demokritos.gr (S.X.); 2 Institute of Chemical Biology, National Hellenic Research Foundation, 11635 Athens, Greece; 3 Institute of Biosciences and Applications, National Center for Scientific Research “Demokritos”, 15341 Athens, Greece; bmavroidi@bio.demokritos.gr; 4 A’ Pathology Department, 401 General Military Hospital of Athens, 11525 Athens, Greece; dr.nikoskalogeropoulos@gmail.com; 5 ICMUB, UMR 6302 CNRS-UB, Université Bourgogne Franche-Comté, 21000 Dijon, France; Franck.Denat@u-bourgogne.fr; 6 Institut UTINAM, UMR 6213 CNRS-UBFC, Université Bourgogne Franche-Comté, 25030 Besançon, France; gautier.laurent@univ-fcomte.fr (G.L.); rana.bazzi@univ-fcomte.fr (R.B.); stephane.roux@univ-fcomte.fr (S.R.)

**Keywords:** actinium-225, alpha emitters, gold nanoparticles, radiolabeling, brachytherapy, cancer therapy

## Abstract

Actinium-225 (^225^Ac) is receiving increased attention for its application in targeted radionuclide therapy, due to the short range of its emitted alpha particles in conjunction with their high linear energy transfer, which lead to the eradication of tumor cells while sparing neighboring healthy tissue. The objective of our study was the evaluation of a gold nanoparticle radiolabeled with ^225^Ac as an injectable radiopharmaceutical form of brachytherapy for local radiation treatment of cancer. Au@TADOTAGA was radiolabeled with ^225^Ac at pH 5.6 (30 min at 70 °C), and in vitro stability was evaluated. In vitro cytotoxicity was assessed in U-87 MG cancer cells, and in vivo biodistribution was performed by intravenous and intratumoral administration of [^225^Ac]^225^Ac-Au@TADOTAGA in U-87 MG tumor-bearing mice. A preliminary study to assess therapeutic efficacy of the intratumorally-injected radio-nanomedicine was performed over a period of 22 days, while the necrotic effect on tumors was evaluated by a histopathology study. We have shown that [^225^Ac]^225^Ac-Au@TADOTAGA resulted in the retardation of tumor growth after its intratumoral injection in U87MG tumor-bearing mice, even though very low activities were injected per mouse. This gold nanoparticle radiopharmaceutical could be applied as an unconventional brachytherapy in injectable form for local radiation treatment of cancer.

## 1. Introduction

Radiation therapy, in conjunction with chemotherapy and surgery, is an effective cancer treatment option, especially for radiation-sensitive tumors. This method, which utilizes high-dose ionizing radiation to kill cancer cells and prevent progression and recurrence of the tumor, falls into one of three categories: external radiation, systemic radiation therapy and internal radiation. External radiation therapy delivers high-energy X-rays or electron or proton beams to a tumor from outside the body, often under imaging guidance. Systemic radiation therapy delivers soluble radioactive substances, either by ingestion, catheter infusion, or intravenous administration of tumor-targeting carriers (such as antibodies or biocompatible materials) that carry selected radioisotopes. Internal radiation therapy (also called brachytherapy) places radiation sources within or near the tumor using minimally invasive procedures. The skillful, precise and targeted characteristics of brachytherapy serve a number of key advantages for the efficacious treatment of solid tumors such as a decrease in side effects, shortened treatment times and cost-effectiveness. Brachytherapy devices have given promising results in preclinical and clinical studies. However, they require a complicated implantation technique under general anesthesia. Furthermore, seed migration may also occur after implantation, and seed removal can be required [1].

Nanoparticles can be manufactured to directly deliver radiation dose to the tumor like brachytherapy; thus, such a technology using nanoparticles is called nanobrachytherapy [2,3,4]. In this perspective, radioactive nanoparticles could represent a promising alternative to current brachytherapy methods with outstanding results compared to conventional brachytherapy. A recent example of this approach is the work by Laprise-Pelletier et al., who report on a new brachytherapy procedure involving intratumoral injections of radioactive Au NPs as a form of brachytherapy for prostate cancer [5]. Herein, we propose a novel radiation nanomedicine that can be applied as unconventional brachytherapy in injectable form, after local intratumoral injection of gold nanoparticles labeled with an alpha (α-) emitter. This type of injectable system should sustain the advantages of brachytherapy while making system administration easier, less invasive (injection instead of implantation) and patient-tailored (splitting of doses into several parts). Furthermore, injectable radiopharmaceuticals do not require seed removal, thus decreasing the difficulty of handling and making them extremely useful. Their nanometer size may permit local diffusion from the injection site, thus further homogenizing radiation deposition within the tumor. Finally, gold nanoparticles are useful multifunctional carriers that not only deliver radioisotopes but also provide imaging and therapy capabilities, since they can be used as contrast agents and can be utilized for photothermal therapy of cancer.

Alpha particles are highly cytotoxic agents, which deposit the whole of their energy within a few cell diameters (50–100 μm). Actinium-225 (^225^Ac, *t*_1/2_ = 9.9 days) is a highly promising α-emitter with a wide range of applications in radiotherapy of cancer mainly due to its long half-life that is well-matched for use in combination with targeting molecules, such as antibodies or nanoparticles. ^225^Ac-labeled molecules have attracted increasing attention the last few years because of their outstanding properties [6]. Among these properties is the ability to induce significantly more double-strand breaks to a DNA molecule than beta (β)-particles, meaning that they are more cytotoxic [7], do not depend upon hypoxia or cell cycle considerations [8,9] and have a relatively low gamma (γ)-ray component in their decay, allowing for outpatient treatments and lower radiation doses to nuclear medicine personnel [10]. As a result, the utilization of ^225^Ac-labeled molecules ensures high biological effectiveness with low-dose radiation.

Under this scope, our aim in the current study was the development and evaluation of a gold nanoparticle radiolabeled with the α-emitter ^225^Ac via a DOTA-derivative chelator as an injectable radiopharmaceutical form of brachytherapy (BRT) seeds for local radiation treatment of cancer. The obtained radio-nanomedicine, mentioned henceforth as [^225^Ac]^225^Ac-Au@TADOTAGA, was biologically evaluated for its stability and in vitro toxicity in glioblastoma U-87 MG cells. Biodistribution in U-87 MG tumor-bearing SCID mice was studied at different time-points, after both intravenous and intratumoral injection of the radiotracer. Finally, therapeutic efficacy was also assessed by carrying out tumor regression studies in mice bearing subcutaneous U-87 MG tumors, after intratumoral injection of [^225^Ac]^225^Ac-Au@TADOTAGA, over a period of 22 days.

## 2. Materials and Methods

***Warning!*** *The ^225^Ac isotope present serious health threats and requires special radioprotective precautions during handling to reduce the risk of harm. This research was conducted in a radiological facility which has all the necessary infrastructure, expertise and license to safely conduct experiments with radioisotopes.*

Materials used for the synthesis of gold nanoparticles were purchased from Sigma-Aldrich (Lyon, France) and CheMatec (Dijon, France). The buffer used for radiolabeling was prepared from trace-free reagents (Sigma-Aldrich, Munich, Germany). U-87 MG (human glioblastoma–astrocytoma) cell line was acquired from the cell bank of the Laboratory of Radiobiology, Institute of Nuclear & Radiological Sciences and Technology, Energy & Safety, NCSR “Demokritos” (Athens, Greece). Cells were free of mycoplasma contamination, as judged visually under microscope observation and by regular 4′,6-diamidine-2′-phenylindole dihydrochloride (DAPI) staining of the cell cultures. The media for the cultures were purchased from Biowest (Nuaillé, France) and the MTT reagent (3-(4,5-dimethylthiazol-2-yl)-2,5-diphenyl-tetrazolium bromide) was obtained from Applichem (Darmstadt, Germany). Optical density measurements in in vitro experiments were conducted using a Spark Multimode Microplate Reader (Tecan, Männedorf, Switzerland) and a LabSystems Multiskan RC Microplate Reader (Thermo Fisher Scientific, Waltham, MA, USA) for the non-radiolabeled and radiolabeled complex, respectively. Actinium-225 was purchased from ITG Garching (Garching, Germany). All other reagents and solvents used in these studies were obtained from commercial sources without further purification. Radioactivity of the [^225^Ac]AcCl_3_ and the radiolabeled nanoparticles was measured using a dose calibrator (Capintec, Ramsey, NJ, USA). Thin-layer chromatography (TLC) silica gel 60 sheets (5 × 10 cm) were purchased from Merck (Dermstadt, Germany) and along with a Radio-TLC Scanner (Scan-Ram, LabLogic, Sheffield, UK) were used in the determination of radiolabeling yield/purity and in vitro stability studies. Purification of the radiolabeled nanoparticles was performed with Amicon Ultra 2 mL centrifugal filters, 50.000 NMWL (Merck Millipore Ltd., Cork, Ireland). Water was deionized to 18 MΩ·cm using an easy-pure water filtration system (Barnstead International, Dubuque, IA, USA). A gamma scintillation counter, Cobra II (Canberra, Packard, Downers Grove, IL, USA) was used to measure the radioactivity of each organ and blood samples in ex vivo biodistribution studies.

Animals used for the biodistribution studies were obtained from the breeding facilities of the Institute of Biosciences and Applications, NCSR “Demokritos”. Our experimental animal facility is registered according to the Greek Presidential Decree 56/2013 (Reg. Number: EL 25 BIO 022), in accordance to the European Directive 2010/63 which is harmonized with national legislation, on the protection of animals used for scientific purposes. All applicable national guidelines for the care and use of animals were followed. The study protocol was approved by the Department of Agriculture and Veterinary Service of the Prefecture of Athens (Protocol Number: 1607/11-04-2018).

### 2.1. Synthesis of Macrocycle-Coated Gold Nanoparticles

The syntheses were adapted from the single-phase protocol developed by Brust et al. [11]. The reduction of the gold salt (HAuCl_4_·3H_2_O) by NaBH_4_ yields gold nanoparticles in presence of ligands whose adsorption on growing particles ensures the control of the size and the colloidal stability.

For a typical preparation of Au@TADOTAGA gold nanoparticles, 50 mg (1.22 × 10^−4^ mol) of HAuCl_4_·3H_2_O, dissolved in 20 mL of methanol, was placed in a 250 mL round-bottom flask. TADOTAGA (86 mg, 1.22 × 10^−4^ mol) in 10 mL of water was added to the gold salt solution under stirring. The mixture turned from yellow to orange. After few minutes, an aqueous solution of NaBH_4_ (48 mg, 12.7 × 10^−4^ mol in 3 mL of water) was added to the mixture under vigorous stirring at room temperature (RT). The stirring was maintained for 1 h. Then, the mixture was dialyzed using a 6000–8000 molecular weight cut-off membrane.

#### 2.1.1. UV–Visible Spectroscopy

UV–visible absorption spectra of functionalized gold nanoparticle colloidal suspensions were recorded at RT with a SPECORD 250 spectrophotometer (Analytic Jena AG, Jena, Germany) in the 400–800 nm range. The spectral measurements were performed at different pH on a diluted colloid ([Au] = 5 × 10^−4^ M) introduced in a standard quartz cuvette (10 mm path length).

#### 2.1.2. Hydrodynamic Diameter and Zeta Potential Measurements

Direct determination of the hydrodynamic diameter and zeta potential of nanoparticles was performed with a Zetasizer from Malvern Instruments (Malvern Panalytical, Orsav, France). The suspension was diluted to obtain a concentration in gold of 4 × 10^−4^ M in an aqueous solution (for hydrodynamic diameter measurements) and in NaCl (0.01 M) aqueous solution adjusted to the desired pH (for zeta-potential measurements).

#### 2.1.3. Transmission Electron Microscopy (TEM)

TEM was used to obtain detailed morphological information about the samples and was carried out using a JEOL 2010 microscope (JEOL Europe SAS, Croissy-sur-Seine, France) operating at 200 kV. The samples for TEM were prepared by depositing a drop of a diluted aqueous suspension of Au@TADOTAGA nanoparticles on a carbon grid (carbon film on Cu 300 square mesh, ultrathin coating, Delta Microscopies, Mauressac, France) and allowing the liquid to dry in air at RT.

### 2.2. Radiolabeling of Au@TADOTAGA Gold Nanoparticles with Actinium-225

Au@TADOTAGA gold nanoparticles (100 μL, Au: 5.07 nmol) were added to trace-free sodium acetate buffer 0.2 M (pH 5.6). Then, 10 to 50 kBq of [^225^Ac]^225^AcCl_3_ were added, following which the mixture was slightly vortexed and consequently incubated at 70 °C for 30 min. The reaction mixture was cooled to RT, and the percentage of ^225^Ac incorporated onto the NPs was determined by instant thin-layer chromatography (ITLC) using silica gel impregnated glass fiber sheets and sodium citrate pH 4 as the mobile phase. In this chromatography system, the radiolabeled NPs remain at the origin (*R*_f_ = 0.0), while unbound ^225^Ac migrates to the solvent front (*R*_f_ = 0.8–1.0). The radiolabeled sample was purified by centrifugation (8110 rcf, 15 min) with Amicon Ultra 2 mL centrifugal filters (50.000 NMWL). The % radiochemical purity of [^225^Ac]^225^Ac-Au@TADOTAGA was determined by ITLC, as previously described. A control test was also performed, in the absence of Au@TADOTAGA. The radioactivity on the ITLC-SG strips was visualized using a Radio-TLC Scanner.

### 2.3. In Vitro Stability Studies of [^225^Ac]^225^Ac-Au@TADOTAGA

*In vitro* stability studies were performed using purified [^225^Ac]^225^Ac-Au@TADOTAGA in the presence of phosphate-buffered saline (PBS) of pH 7.4 and sodium acetate buffer of pH 5.6 at RT. For both stability tests, a sample of 10 μL of [^225^Ac]^225^Ac-Au@TADOTAGA was incubated with 90 μL of either PBS or sodium acetate at RT. Aliquots were taken from the mixtures up to 10 days after and analyzed by ITLC as described above. Both experiments were performed in triplicate, from three independent radiolabeling procedures.

### 2.4. Cell Cultures

U-87 MG cells were grown in Dulbecco’s modified Eagle’s medium (DMEM) growth medium of pH 7.4, supplemented with 10% FBS, 100 U/mL of penicillin, 2 mM glutamine, and 100 μg/mL of streptomycin. Cell cultures were maintained in flasks and were grown at 37 °C in a humidified atmosphere of 5% CO_2_ in air. Subconfluent cells were detached using a 0.05% (*w*/*v*) trypsin-0.25% (*w*/*v*) ethylenediaminetetraacetic acid (EDTA) solution, and the subcultivation ratio was 1:2–1:5.

### 2.5. MTT Toxicity Assay

In vitro cytotoxicity of Au@TADOTAGA and [^225^Ac]^225^Ac-Au@TADOTAGA complex against U-87 MG cells was determined by the MTT colorimetric assay. Briefly, cells were seeded in 96-well plates (25 × 10^3^ and 1 × 10^4^ cells/well for the 24 and 48 h incubation times, respectively) and grown overnight at 37 °C in a 5% CO_2_ incubator. Appropriate amounts of the Au@TADOTAGA and [^225^Ac]^225^Ac-Au@TADOTAGA complexes (stock concentration of 20 μg [Au]/mL and the same concentration of gold with an activity of 2 kBq/mL, in DMEM) were added to achieve the desired final concentrations (0.625, 1.25, 2.5, 5, 10 and 20 μg [Au]/mL for the non-radiolabeled complex and 0.0625, 0.125, 0.25, 0.5, 1 and 2 kBq/mL for the radiolabeled complex). The medium was then removed and replaced with 100 μL of MTT solution (1 mg/mL). After a 4 h incubation, the solution was aspirated, formazan crystals were solubilized in 100 μL of isopropanol and absorbance was recorded at 540 nm. The results were expressed as % cell viability = (mean optical density (OD) of treated cells/mean OD of untreated cells) × 100.

### 2.6. Ex Vivo Biodistribution Studies of [^225^Ac]^225^Ac-Au@TADOTAGA

For experimental tumor models, female SCID mice (8-week-old) were inoculated with 1 × 10^7^ U-87 MG glioblastoma cells into the right front leg in 100 μL fetal bovine serum free medium. Biodistribution studies were initiated approximately 12 days after cancer cell inoculation, when the tumors had reached a volume of 200–400 mm^3^. Tumor-bearing mice were randomly separated into two groups (*n* = 3–5 mice/time-point/group). In the first group, [^225^Ac]^225^Ac-Au@TADOTAGA was administered intravenously via the tail vein, while in the second the administration was conducted by intratumoral injection (100 μL, ~1 kBq per mouse for both groups). At 2, 4, 24, 72 and 120 h post-injection (and at 288 h post-injection for the second group), all mice were euthanized and their major organs and tissues were removed, weighed and counted in an automatic γ-counter together with samples of muscles and urine. All measurements were corrected for background and radioactive decay. Tissue distribution data were calculated as the percent injected dose per gram (% IA/g), using an appropriate standard. The stomach and intestines were not emptied before the measurements. The % IA in whole blood was estimated assuming a whole blood volume of 6.5% of the total body weight.

### 2.7. Therapeutic Efficacy Studies

Therapeutic efficacy studies were performed in SCID mice bearing subcutaneous U-87 MG tumor xenografts when the tumor reached a volume of about 300 mm^3^ (about 12 days after inoculation of glioma cells). Mice were randomly divided into two groups (*n* = 5 mice per group) and received three intratumoral injections of either normal saline (control group, 100 μL saline) or [^225^Ac]^225^Ac-Au@TADOTAGA (therapy group, 100 μL NPs/ 5 kBq) (days of injection designated as Day 1, Day 3 and Day 5). Tumor volume was monitored for 22 days using calipers, and was calculated using the formula (length × width^2^)/2 [12,13]. The tumor growth index (TGI) for both animal groups was calculated by dividing the tumor volume by the initial tumor volume on Day 1, before initiation of intratumoral administration of [^225^Ac]^225^Ac-Au@TADOTAGA (in the therapy group). TGI was plotted vs. treatment time post-injection.

### 2.8. Histopathology Studies

After the therapeutic efficacy study the mice were euthanized and the tumors were surgically removed. The tissues were then fixed in 10% formalin. Each tumor was further dissected into pieces approximately every 1 mm and embedded in paraffin. Using a microtome, each paraffin block was dissected to generate 4 μm sections and each section was stained with hematoxylin and eosin. All the H&E slides per block, containing the total mass of each tumor, were evaluated to calculate the total percentage of necrosis per sample. The pictures of each slide were created using an Olympus U-TVO.5XC-3 microscope, equipped with an Infinity1 Lumenera camera (Ontario, Canada, magnification 100×). The digital analysis of the necrotic areas was performed using the FIJI/Image J software (version 2.0.0-rc-69/1.52p.).

### 2.9. Statistical Analysis

The data are presented as means ± standard deviations (SD). For the cytotoxicity and biodistribution studies, the data were compared using an unpaired *t*-test with a significance level of *p* < 0.05. All analyses were performed using Microsoft Excel software. The *p*-values regarding the analysis of the necrosis were calculated using the unpaired *t*-test with Welch’s correction using Prism 8 software version 8.1.1 (224).

## 3. Results

### 3.1. Synthesis and Characterization of Gold Nanoparticles

**Figure 1:**
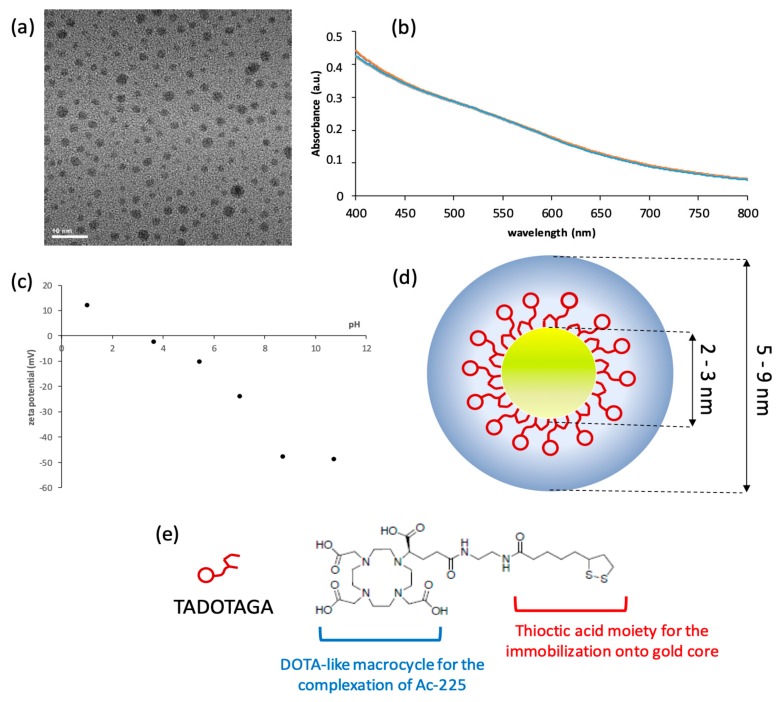
(**a**) TEM micrograph of Au@TADOTAGA nanoparticles; (**b**) UV-visible spectra recorded at pH 3 (blue curve), 5 (red curve), 7 (green curve), 9 (purple curve) and 11 (cyan curve); (**c**) zeta potential of an aqueous suspension of Au@TADOTAGA nanoparticles as a function of pH; (**d**) schematic representation of an Au@TADOTAGA nanoparticle (yellow: gold core; red: TADOTAGA molecules in the organic shell; blue: the immobile solvatation layer); (**e**) chemical structure of TADOTAGA.

The reduction of the gold salt by sodium borohydride in presence of TADOTAGA chelators led to the formation of ultrasmall Au@TADOTAGA gold nanoparticles. These nanoparticles are composed of a gold core with a size ranging from 2 to 3 nm (according to the analysis of TEM micrographs (Figure 1a)) and an organic shell which is composed of TADOTAGA chelators. DLS measurements indicate that the hydrodynamic diameter of Au@TADOTAGA nanoparticles which includes the gold core, the organic shell and the immobile solvatation layer is comprised between 5 and 9 nm (Figure 1d). Therefore, these nanoparticles exhibit suited dimensions for renal clearance. The UV-visible spectra recorded at various pH levels are perfectly superimposed in large pH range (between 3 and 11) (Figure 1b). This observation suggests a high colloidal stability, in particular at the physiological pH (7.4). The colloidal stability can be explained by the electrostatic repulsion between charged Au@TADOTAGA nanoparticles. The measurements of zeta potential from pH 2 to 11 clearly show that Au@TADOTAGA nanoparticles are characterized by a negative zeta potential for pH>4 (Figure 1c). The evolution of the zeta potential with pH can be explained by the presence of ionizable groups in TADOTAGA (Figure 1e). This macrocyclic molecule is composed of four carboxylic acid (-COOH) groups and four ternary amine (NR_1_R_2_R_3_)) functions which are all protonated (-COOH and N^+^HR_1_R_2_R_3_) at low pH. Owing to the positive charge of ammonium groups (N^+^HR_1_R_2_R_3_), the Au@TADOTAGA nanoparticles are positively charged at low pH: zeta potential is therefore positive for pH<2. When pH increases, protons are progressively released from carboxylic acid and ammonium groups. Therefore, the deprotonation provides non-charged amine functions and negatively charged carboxylate moieties. As a result, the Au@TADOTAGA nanoparticles are negatively charged for pH>4. The TADOTAGA chelators were designed for playing at least three important roles: (i) the presence of two sulfur atoms ensures the strong immobilization onto the gold cores and therefore permits the control of particle growth during the synthesis; (ii) the hydrophilic character and the negative charges at physiological pH confer to the gold nanoparticles a high colloidal stability in aqueous media; and (iii) the chelating properties of the DOTA-like macrocycle can be exploited for the immobilization of ions of interest for imaging and/or therapy.

### 3.2. Radiolabeling of Au@TADOTAGA Gold Nanoparticles with Actinium-225

AuNPs bearing the TADOTAGA chelator were radiolabeled after 30 min incubation at 70 °C. Radiochemical yield was assessed by ITLC and was found to be 86% ± 1.8%. Heating up to 2 h did not improve the radiolabeling yield. Therefore, [^225^Ac]^225^Ac-Au@TADOTAGA NPs were subjected to purification by centrifugation with ultracentrifugation filters (recovery yield of radiolabeled NPs after ultracentrifugation: 68.5% ± 2.3%). TLC assessment showed radiochemical purity of the radiolabeled AuNPs of >93%. The sample was diluted with acetate buffer pH 5.6, for biodistribution experiments.

### 3.3. In Vitro Stability Studies

In order to assess the in vitro stability of [^225^Ac]^225^Ac-Au@TADOTAGA in various solutions, the radiolabeled sample was incubated with PBS and sodium acetate buffer pH 5.6 at RT, and stability was monitored over 10 d. The results demonstrated satisfactory in vitro stability in both solutions (~78% stable [^225^Ac]^225^Ac-Au@TADOTAGA in PBS and ~84% in sodium acetate buffer, at 10 days post-incubation) as evaluated by TLC analysis.

### 3.4. In Vitro Cytotoxicity Study of Au@TADOTAGA/[^225^Ac]^225^Ac-Au@TADOTAGA

**Figure 2:**
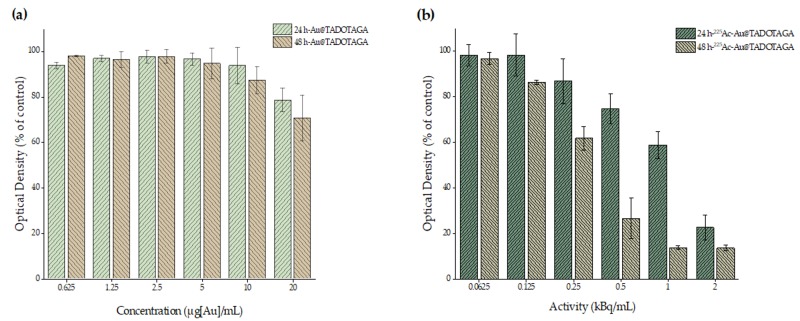
In vitro cytotoxicity of Au@TADOTAGA (**a**) and [^225^Ac]^225^Ac-Au@TADOTAGA (**b**) in U-87 MG cells by MTT assay. Data are presented as mean ± SD (*n* = 3 for **a** and *n* = 2 for **b**).

Figure 2 summarizes the results of the cell toxicity studies in U-87 MG cell line after 24 and 48 h of exposure to different concentrations of Au@TADOTAGA and [^225^Ac]^225^Ac-Au@TADOTAGA complexes. As can been seen in Figure 2a, after 24 and 48 h of exposure with Au@TADOTAGA, no major changes in the cell viability were recorded at concentrations up to 10 µg/mL. At the highest concentration (20 µg[Au]/mL), a reduction of approximately 22% for 24 h (viability of 88%) and 20% for 48 h (viability of 80%) on the cell viability was observed. On the other hand, treatment with [^225^Ac]^225^Ac-Au@TADOTAGA (Figure 2b, molar activity of [^225^Ac]^225^Ac-Au@TADOTAGA: 2 kBq/20 μg[Au]) significantly decreases the viability of U-87 MG cells in a concentration-dependent manner after 24 h of incubation, starting from the concentration of 0.5 kBq/mL and reaching a value of 22% (~77% reduction) at the concentration of 2 kBq/mL. A similar trend in a more pronounced manner was observed after 48 h of incubation, since the recorded values of cell viability gradually decreased from 96% to 13% (87% reduction at the highest concentration). Furthermore, the recorded reduction started at a lower concentration (0.25 kBq/mL).

### 3.5. Ex Vivo Biodistribution Studies: Intravenous vs. Intratumoral Injection of [^225^Ac]^225^Ac-Au@TADOTAGA

**Figure 3:**
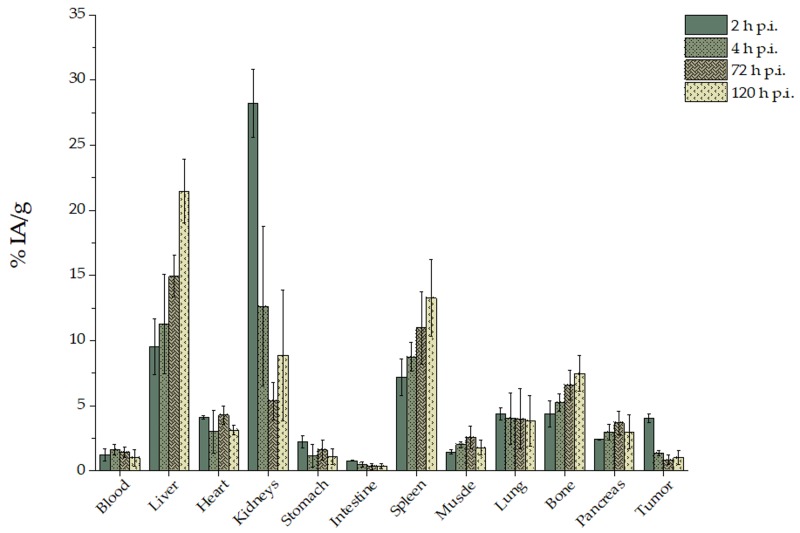
Ex vivo biodistribution of [^225^Ac]^225^Ac-Au@TADOTAGA after intravenous injection. Values represent the mean ± SD (*n* = 5 mice per group).

Intravenous administration of [^225^Ac]^225^Ac-Au@TADOTAGA was monitored over a period of 120 h p.i. (Figure 3). This study demonstrated that the NPs accumulated mainly in the kidneys, liver and spleen. At 2 h p.i, uptake in the kidneys was 28.21% ± 2.64% IA/g, which decreased in time (8.88% ± 5.03% IA/g at 120 h p.i.), thus reflecting renal clearance. Liver and spleen uptake showed an opposite trend, with 9.50% ± 2.14% IA/g and 7.20% ± 1.42% IA/g at 2 h, which at 120 h increased to 21.46% ± 2.44% IA/g and 13.27% ± 2.93% IA/g, respectively. Tumor radioactivity peaked at 2 h p.i. (4.05% ± 0.34% IA/g) and exhibited a 4-fold decrease by 120 h p.i., remaining practically stable after 4 h p.i. Bone uptake showed a gradual increase from 2 to 120 h p.i. (4.38% ± 1.02% to 7.48% ± 1.36% IA/g). No major uptake in other organs is noted.

**Figure 4:**
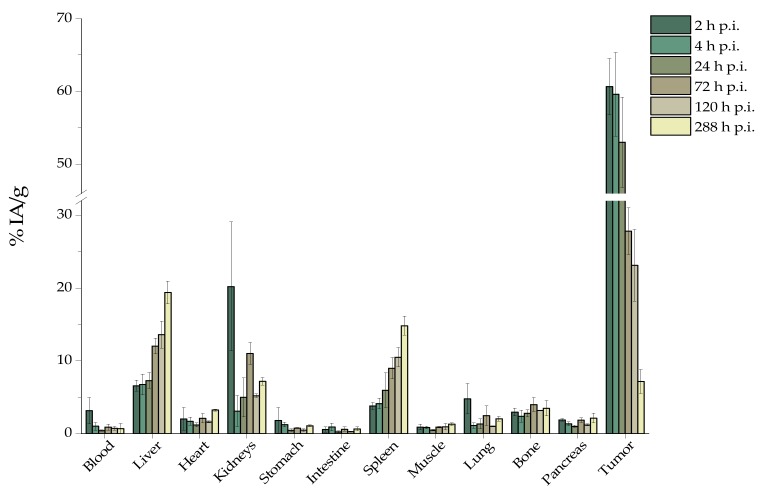
Ex vivo biodistribution of [^225^Ac]^225^Ac-Au@TADOTAGA after intratumoral injection. Values represent the mean ± SD (*n* = 5 mice per group).

Biodistribution studies after intratumoral injection of [^225^Ac]^225^Ac-Au@TADOTAGA resulted in high tumor uptake at 2 h p.i. (60.67% ± 3.87% IA/g) which decreased over 228 h p.i. (5.21% ± 1.26% IA/g). Liver and spleen exhibited an opposite trend, showing ~3-fold and ~4-fold increases from 2 h to 288 h p.i., respectively. Bone uptake was 2.95% ± 0.54% IA/g which increased to 3.48% ± 1.06% IA/g at 288 h p.i. Radioactivity in all other organs was less than 2% IA/g from 4 h p.i., with the exception of kidney uptake (Figure 4).

### 3.6. Therapeutic Efficacy Study

**Figure 5:**
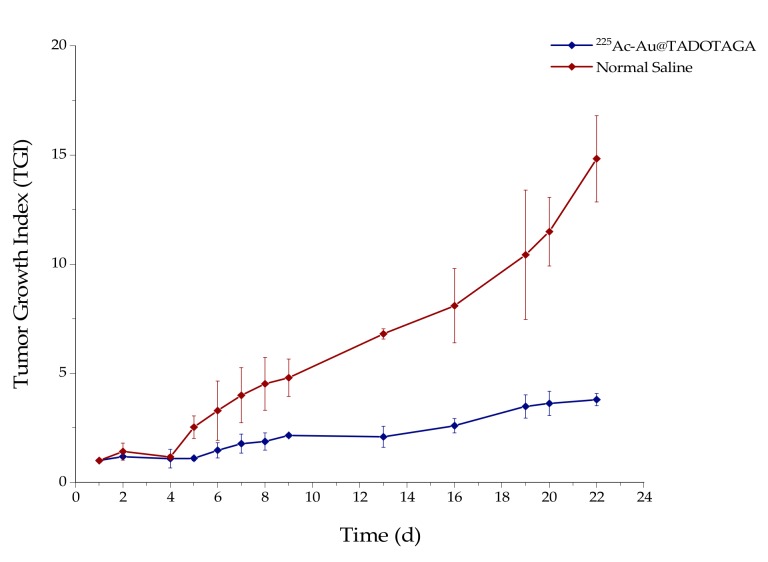
Effect of intratumoral injection of [^225^Ac]^225^Ac-Au@TADOTAGA (blue line) or normal saline (red line) on the tumor growth index (TGI) of U-87 MG tumor-bearing SCID mice. Values represent the mean ± SD (*n* = 5 mice per group).

The therapeutic efficacy of [^225^Ac]^225^Ac-Au@TADOTAGA was determined by estimation of the tumor growth index (TGI) of two groups of U-87 MG tumor-bearing SCID mice, up to 22 days post-treatment by intratumoral injection of NPs (Group A) and normal saline (Group B) (Figure 5, Group A: blue line; Group B: red line). Mice in Group A received three injections of [^225^Ac]^225^Ac-Au@TADOTAGA (total activity: 15 kBq), while mice in Group B received three injections of normal saline, on Day 1 (day of treatment initiation), Day 3 and Day 5. TGI of Group A mice was ~2.4-fold lower at 8 days p.i. and showed an upward trend, reaching ~3.9-fold lower at 22 days p.i., when compared to the mice in Group B.

### 3.7. Histopathology Study

**Figure 6:**
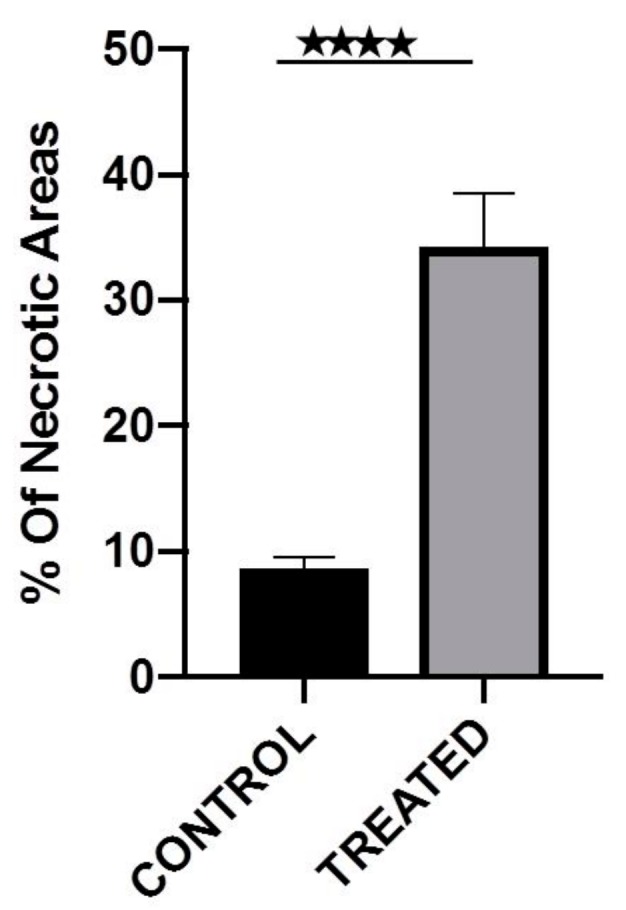
Percentage of the total necrotic areas per tumor. The 4 stars indicate the *p* value. *p* < 0.0001.

**Figure 7:**
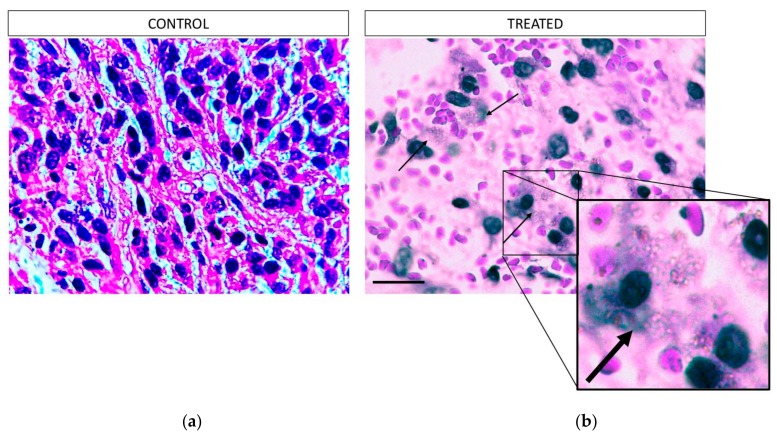
Representative images of H&E-stained slices of U-87 MG tumor tissue after the therapeutic efficacy study in mice, from the area of the necrotic lesion (100× magnification): (**a**) control mice, (**b**) treated mice. The arrows indicate the presence of nanoparticles in periphery of the necrotic lesions of the treated tumors. The box shows a 5× digital zoom of the selected area. Scale bar = 40 μm.

The total tumor and the subsequent necrotic areas were measured per slide (see Section 2). All the H&E slides per block, containing the total mass of each tumor, were evaluated to calculate the total percentage of necrosis per sample (Figure 6). The graph presents the average of the measurements from all the samples per group. From the analysis of the necrotic areas from the treated and the control animals, we reveal that the Au@TADOTAGA gold nanoparticles with Ac-225 caused increased damage to the tumors, resulting in increased tumor tissue necrosis. The tumor-adjacent tissues in both the treated and the control animals revealed no significant necrosis, a fact which is related to the localization of the nanoparticles inside the tumors. Using a 100× magnification (Figure 7) we could observe the presence of the nanoparticles in the periphery of the necrotic lesions.

## 4. Discussion

Actinium-225 is a highly promising alpha-emitter for therapeutic applications due to its favorable nuclear properties and high cytotoxicity, which is attributed to the generation of 4 high-energy α particles during its decay. Nonetheless, the application of ^225^Ac remains challenging, as chelation via most chelating agents is insufficient to stably retain its daughters, due to the α recoil effect which is observed upon release of an α particle. While Thiele et al. recently reported on the development of an eighteen-membered macrocyclic ligand which rapidly and efficiently chelates ^225^Ac at RT, no mention was made with regard to how efficiently this chelator retains the daughter isotopes of ^225^Ac [14]. DOTA is probably not the most suitable chelator for ^225^Ac and its daughters, however for the moment it remains the gold standard for the coordination of ^225^Ac [6]. This is reflected in numerous preclinical studies involving biomolecules radiolabeled with ^225^Ac via DOTA, which have shown promising results for the treatment of various cancers [15,16]. It is also evident in the clinic, where for the past 10 years several clinical trials have been underway with ^225^Ac-DOTA constructs for the treatment of, e.g., prostate and gastroenteropancreatic cancers [17,18].

During the past decade, an increasing number of studies have been reported on tumor treatment with radiolabeled NPs, most of which have been performed with NPs radiolabeled with the β-emitting isotope Lutetium-177 (^177^Lu, half-life: *t*_1/2_ = 6.7 d) [12,19,20,21,22,23,24]. Regarding ^225^Ac, only a few studies have examined NPs radiolabeled with this radionuclide for tumor treatment [25,26,27]. The present work was designed to investigate a gold nanoparticle radiolabeled with an α-emitter as an injectable radiopharmaceutical form of BRT for local radiation treatment of cancer. For this purpose, the accumulation of [^225^Ac]^225^Ac-Au@TADOTAGA NPs in a U-87 MG tumor model, after both intravenous and intratumoral injection, was studied. Ex vivo data are provided up to 120 h post-injection, while data from one extra time-point are provided from the intratumorally-injected mice of the therapeutic efficacy study, after their euthanization at 288 h p.i. Our present work demonstrates the therapeutic potential of [^225^Ac]^225^Ac-Au@TADOTAGA after their intratumoral injection in glioma xenografts. To our knowledge, this is the first long-term biodistribution study performed with ^225^Ac-labeled gold nanoparticles.

Au@TADOTAGA nanoparticles were efficiently labeled with ^225^Ac by heating at 70 °C for 30 min, resulting in a radiochemical yield of 86% ± 1.8%. After purification by centrifugation, the radiochemical purity was ~93%. The radiolabeled NPs were stable up to ~80% in both PBS and acetate buffer pH 6.5 at 10 days post-incubation.

In order to assess the cytotoxic properties of the new Au@TADOTAGA and [^225^Ac]^225^Ac-Au@TADOTAGA complexes, cell toxicity studies with the MTT assay were conducted with the use of glioblastoma U-87 MG cell line. The experimental data revealed that Au@TADOTAGA showed no remarkable cytotoxicity at 24 and 48 h after treatment. However, in the case of 24 h treatment with [^225^Ac]^225^Ac-Au@TADOTAGA, a dose-dependent cytotoxicity pattern was observed that can only be attributed to the presence of ^225^Ac. A similar trend is also present at 48 h at an even lower concentration (0.25 instead of 0.5 kBq/mL), additionally demonstrating a time-dependent manner of [^225^Ac]^225^Ac-Au@TADOTAGA-induced toxicity in U-87 MG cells.

Ex vivo biodistribution studies of [^225^Ac]^225^Ac-Au@TADOTAGA were performed on U-87 MG tumor-bearing SCID mice, up to 120 h after intravenous injection, and 288 h after intratumoral injection. After their intravenous injection, [^225^Ac]^225^Ac-Au@TADOTAGA NPs were mainly located in the kidneys, liver and spleen. Initial high kidney uptake demonstrates route of clearance, as noted in the literature for such gold NPs [28,29,30]. Tumor uptake peaked at 2 h p.i. and is due to the enhanced permeability and retention (EPR) effect, which results in the accumulation of these NPs within the tumor because of the tumor’s leaky vasculature and poor lymphatic drainage. Thereafter, tumor uptake declined while liver and spleen uptake showed an upward trend. This may be attributed to two factors: (i) recirculation of the radiolabeled NPs in the mouse organism and consequent clearance from the body via the hepatobiliary route and (ii) slow and gradual release of the ^225^Ac radiolabel from the chelator [15,31,32]. This is also evident in the decline in tumor-to-tissue ratios over time (tumor:blood 3.66 ± 1.83 vs. 1.41 ± 1.24; tumor:liver 0.45 ± 0.14 vs. 0.05 ± 0.02; tumor:spleen 0.58 ± 0.17 vs. 0.09 ± 0.07). The moderate liver uptake at early time-points (2 and 4 h p.i.) is unlikely to be due to the phenomenon of opsonization, as this would lead to fast and pronounced uptake of the NPs by phagocytic-rich organs such as the liver and spleen [33,34]. Regarding tumor uptake, our results showed satisfactory tumor uptake via passive targeting, comparable to results presented in the literature [35,36]. The observed bone uptake of [^225^Ac]^225^Ac-Au@TADOTAGA (4.38% ± 1.02% IA/g at 2 h p.i., increasing to 7.48% ± 1.36% IA/g at 120 h p.i.) may be attributed to partial release of the radiolabel over time [15].

After intratumoral injection, we observed a very high tumor uptake, which slowly decreased over the duration of the study (2 to 288 h p.i.). On the contrary, radioactivity in the liver and spleen significantly increased (liver uptake: 6.57% ± 0.75% IA/g and 19.37% ± 1.56% IA/g at 2 and 288 h p.i., *p* = 0.012; spleen uptake: 3.80% ± 0.48% IA/g and 14.80% ± 1.30% IA/g at 2 and 288 h p.i., *p* = 0.0108). This behavior can be attributed to recirculation of the radiolabeled NPs, as well as to partial release of ^225^Ac from the chelator, which was also demonstrated during our in vitro stability studies [15,31,32]. Although [^225^Ac]^225^Ac-Au@TADOTAGA NPs do not bear a targeting vector, high tumor retention was provided after intratumoral injection. Recently, Yook et al. reported on the radiolabeling of both nontargeted and targeted Au NPs with Lutetium-177 and showed that both radiolabeled NPs showed high tumor uptake after intratumoral injection, which slowly cleared over 48 h [12]. Similar results on active and passive accumulation of gold nanomaterials have been reported [37,38].

While uptake of NPs by the RES organs is always an issue when these are systemically administered, previous results with Au@TADOTAGA radiolabeled with Indium-111 showed low to moderate liver and spleen uptake, which is in accordance to our results [28]. This study revealed a similar distribution of the radiolabeled NPs, however the NPs were monitored up to 72 h post intravenous injection. The present work provides in vivo data up to 120 h after intravenous injection and 288 h after intratumoral injection of these NPs radiolabeled with ^225^Ac in U-87 MG tumor-bearing mice. A major difference between our study and the study by Laurent et al. is the high tumor uptake of [^225^Ac]^225^Ac-Au@TADOTAGA after intratumoral administration when compared to their intravenous administration, which is expected [28].

As proof-of-concept, a preliminary study to assess the therapeutic effect of intratumorally-injected [^225^Ac]^225^Ac-Au@TADOTAGA was performed over a period of 22 days. Our results suggest that the intratumoral route of administration was responsible for delaying tumor growth, even though the amount of radiotracer injected per mouse was low (~15 kBq over three i.t. injections). On the contrary, mice injected with normal saline exhibited more rapid tumor growth. Histopathology studies confirm our in vivo studies.

## 5. Conclusions

Gold nanoparticles radiolabeled with ^225^Ac via the macrocyclic chelator TADOTAGA resulted in the retardation of tumor growth after their intratumoral injection in U87MG tumor-bearing mice, even though very low activities were injected per mouse. To our knowledge, this is the first such study reported in the literature. This gold nanoparticle radiopharmaceutical could be applied as an unconventional brachytherapy in injectable form for local radiation treatment of cancer. In order to improve their properties, these AuNPs will be further derivatized with other ligands for ^225^Ac chelation, in order to maximize their in vivo stability, which is particularly important for long-term in vivo studies with an α-emitter. Further preclinical evaluation will include dose-escalation studies of [^225^Ac]^225^Ac-AuNPs in tumor-bearing mice, to determine whether or not this will lead to an improvement in the therapeutic effect. We aspire that this agent could also exploit the beneficial radiosensitizing effect of the gold core, enhancing therapeutic efficiency of brachytherapy.
